# Silibinin induces oral cancer cell apoptosis and reactive oxygen species generation by activating the JNK/c-Jun pathway

**DOI:** 10.7150/jca.84734

**Published:** 2023-06-26

**Authors:** Haibo Zhang, Hyeonjin Kim, Si-Yong Kim, Huang Hai, Eungyung Kim, Lei Ma, Dongwook Kim, Chae Yeon Kim, Kanghyun Park, Sijun Park, Jiwon Ko, Eun-Kyong Kim, Kirim Kim, Zae Young Ryoo, Junkoo Yi, Myoung Ok Kim

**Affiliations:** 1 Department of Animal Science and Biotechnology, Research Center for Horse industry, Kyungpook National University, Sangju-si, Republic of Korea.; 2 College of Pharmacy, Henan University of Chinese Medicine, Zhengzhou, 450046, China.; 3 School of Life Sciences, BK21 FOUR KNU Creative BioResearch Group, Kyungpook National University, Daegu, Korea.; 4 Department of Dental Hygiene, Kyungpook National University, Sangju, Republic of Korea.; 5 School of Animal Life Convergence Science, Hankyong National University, Anseong, 17579, Republic of Korea.

**Keywords:** Silibinin, ROS, JNK, Oral cancer, Xenograft

## Abstract

**Background:** Oral cancer is one of the most prevalent malignant tumors worldwide. Silibinin has been reported to exert therapeutic effects in various cancer models. However, its mechanism of action in oral cancer remains unclear. We aimed to examine the molecular processes underlying the effects of silibinin in oral cancer *in vitro* and *in vivo* as well as its potential anticancer effects. Next, we investigated the molecular processes underlying both in vitro and in vivo outcomes of silibinin treatment on oral cancer.

**Methods:** To investigate the effects of silibinin on the growth of oral cancer cells, cell proliferation and anchorage-independent colony formation tests were conducted on YD10B and Ca9-22 oral cancer cells. The effects of silibinin on the migration and invasion of oral cancer cells were evaluated using transwell assays. Flow cytometry was used to examine apoptosis, cell cycle distribution, and accumulation of reactive oxygen species (ROS). The molecular mechanism underlying the anticancer effects of silibinin was explored using immunoblotting. The *in vivo* effects of silibinin were evaluated using a Ca9-22 xenograft mouse model.

**Results:** Silibinin effectively suppressed YD10B and Ca9-22 cell proliferation and colony formation in a dose-dependent manner. Moreover, it induced cell cycle arrest in the G0/G1 phase, apoptosis, and ROS generation in these cells. Furthermore, silibinin inhibited the migration and invasion abilities of YD10B and Ca9-22 cells by regulating the expression of proteins involved in the epithelial-mesenchymal transition. Western blotting revealed that silibinin downregulated SOD1 and SOD2 and triggered the JNK/c-Jun pathway in oral cancer cells. Silibinin significantly inhibited xenograft tumor growth in nude mice, with no obvious toxicity.

**Conclusions:** Silibinin considerably reduced the development of oral cancer cells by inducing apoptosis, G_0_/G_1_ arrest, ROS generation, and activation of the JNK/c-Jun pathway. Importantly, silibinin effectively suppressed xenograft tumor growth in nude mice. Our findings indicate that silibinin may be a promising option for the prevention or treatment of oral cancer.

## Introduction

Oral cancer is a common malignancy of the head and neck region 1, with more than 350,000 new cases and 177,000 deaths annually recorded worldwide 2. Tobacco and alcohol use increase the development and incidence rates of oral cancer as the main risk factors. Although treatment for oral cancer has improved over the years, the disease still proves fatal to a large number of patients. Surgery is the mainstay of treatment for oral cancer, and patients with advanced-stage or inoperable cancer are usually treated with combination therapy 3. Drug resistance and adverse side effects are the complications associated with oral cancer chemotherapy. Hence, finding more effective and safer drugs and developing novel therapies for oral cancer is of great theoretical and practical significance.

Reactive oxygen species (ROS) induce oxidative stress and are involved in apoptosis and autophagy. 4, 5. ROS are regarded as crucial elements in tumor development. 6. Increased ROS levels induce cancer cell death by regulating the c-Jun N-terminal kinase (JNK)/c-Jun pathway 4, 7. JNKs are major protein kinases that control a variety of physiological processes, such as inflammation, morphogenesis, cell proliferation, differentiation, survivability, and apoptosis. JNK activation triggers apoptotic mechanisms, such as Bcl-2 and Bcl-xl inhibition, along with Bax activation. 8, 9. Increasing evidence suggests that the JNK pathway exhibits tumor-suppressive functions in cancer. 10, 11. These findings indicate that targeting the ROS/JNK pathway could serve as an effective cancer treatment strategy.

Silibinin, a flavonolignan isolated from *Silybum marianum* L. Gaertn., has been extensively utilized in the treatment of liver disorders and detoxification 12-14. It exerts anti-cancer effects against many cancer types 15, including breast 16, 17, cervical 18, liver 19, 20, and oral cancers 21, and promotes the formation of mitochondrial NOS, which induces apoptosis in human epidermal carcinoma A431 cells. 22. Furthermore, silibinin reduces prostate cancer cell migration and invasion by inhibiting the expression of vimentin and MMP-2 23, and induces apoptosis and G1 arrest in pancreatic cancer cells 24. To date, only two studies have been conducted on the effect of silibinin on oral cancer, which support the hypothesis that silibinin inhibits SSC-4 oral cancer cell proliferation and invasion by inhibiting the MAPK pathway 21 and triggers SSC-25 oral cancer cell apoptosis through the mitochondrial pathway 25. Although these studies suggest that silibinin has potent anticancer effects, the mechanisms underlying these effects in oral cancer have not been fully explored.

Current research has focused on the anti-cancer properties of silibinin in oral cancer. We found that these effects were mediated by the induction of apoptosis and ROS generation through activation of the JNK/c-Jun pathway.

## Materials and methods

### Reagents and antibodies

Silibinin (purity >98%, as assessed by high-performance liquid chromatography) and N-acetyl-L-cysteine (NAC) were procured from Sigma-Aldrich (St. Louis, MO, USA). Dimethyl sulfoxide (DMSO) was used to dissolve silibinin during in vitro experiments. Primary antibodies against Bcl-2 (#3498), cleaved PARP (#5625), Bax (#5023), cyclin D1 (#2922), cleaved caspase-3 (#9661), cyclin-dependent kinase4 (CDK4) (#12790), CDK6 (#3136), cyclin E1 (#4129), E-cadherin (#3195), N-cadherin (#4061), , p-AKT (#9271), AKT (#9272), p-ERK (#9101), ERK (#9102), p-p38 (#9216), p38 (#9212), cyclin D1 (#2922), p-c-Jun (#3270S), and c-Jun (#9165) were procured from Cell Signaling Technology (Danvers, MA, USA). And p53 (sc-1312), SOD1 (sc-11407), SOD2 (sc-18503), β-Actin (sc-47778) was obtained from Santa Cruz Biotechnology (Santa Cruz, CA, USA). Also, vimentin (ab92547) was obtained from abcam.

### Cell culture

Ca9-22 (Japanese Collection of Research Bioresources Cell Bank, Shinjuku, Japan) and YD10B (Oral Cancer Institute of the College of Dentistry at Yonsei University, Seoul, Republic of Korea) 26 cells were cultured in Dulbecco's modified Eagle's medium supplemented with 10% fetal bovine serum (FBS; Gibco) and 1% penicillin/streptomycin. All the cells were grown in a 5% CO_2_ incubator at 37°C 27.

### Cell viability assay

The Cell Counting Kit-8 (CCK-8; Dojindo Laboratories, Kumamoto, Japan) assay was used to assess cell viability. Briefly, oral cancer cells were plated in 96-well plates (1×10^3^ cells/well) to allow attachment and were incubated overnight. Cells were treated with 0, 50, 100, and 200 μM silibinin for 0, 24, 48, 72, and 96 h, and then incubated with 10 µL CCK-8 solution in each well for 1 h at 37°C in an atmosphere of 5% CO_2_. A spectrophotometer was used to measure absorbance at 450 nm (BioTek) 27.

### Anchorage-independent cell growth

Oral cancer cells (8 × 10^3^ cells/well) were seeded in complete growth medium containing 0.3% agar and different concentrations of silibinin (0, 50, 100, and 200 μM) and then overlaid onto 6-well plates containing 0.6% agar base and specified concentrations of silibinin. The plates were incubated at 37°C in an atmosphere of 5% CO_2_ for 2 weeks, followed by which they were photographed under a microscope (Leica, Wetzlar, Germany), and ImageJ was used to count the colonies 27.

### Migration and invasion assays

Transwell plates were used for the cell migration and invasion tests. (Corning, NY, USA) according to the manufacturer's instructions. The lower compartment was filled with 600 μl medium containing 10% FBS. Samples containing 8 × 10^4 ^cells in 100 μl medium containing 1% FBS were added to the upper chamber. After 12 h, different concentrations of silibinin were added to the upper chamber. After incubation for 48 h, the cells were fixed with methanol for 15 min, and a cotton swab used to remove the noninvasive cells. Invading cells were stained with 0.1% crystal violet. For invasion assays, the chamber was pre-coated with Matrigel and 8 × 10^4^ cells were plated onto the upper chamber. The other steps were performed in the same manner as the migration assay. The number of invading cells was quantified by counting stained cells under a microscope.

### Cell cycle and apoptosis analysis

Oral cancer cells were seeded in 60-mm culture dishes (1 × 10^5^ cells/dish). After incubation for 12 h, the cells were treated with different concentrations of silibinin (0, 50, 100, and 200 μM) for 48 h. Cells were collected, fixed in 70% cold ethanol, and stored at -20°C overnight for cell cycle analysis. The cells were then centrifuged (3000 rpm, 5 min), incubated with RNase (100 μg/ml) in phosphate-buffered saline (PBS) for 30 min at 37°C, and stained with propidium iodide (PI; 20 μg/ml) for another 30 min in the dark. Cells were collected and stained with Annexin V (BioLegend, San Diego, CA, USA) and PI for the apoptosis test. FACS Verse Flow Cytometry (BD Biosciences, CA, USA) was used to examine the cell cycle distribution and apoptosis.

### Measurement of ROS levels

Intracellular ROS levels were determined using 2, 7-dichlorofluorescein diacetate. (DCFH-DA) staining. Briefly, cells were seeded into 60-mm culture dishes (1 × 10^5^ cells/dish). After incubation for 12 h, cells were treated with different concentrations of silibinin (0, 50, and 200 μM) for 48 h, washed with PBS, and incubated for 30 min with 10µM DCFH-DA in FBS-free medium at 37°C. The cells were then collected and resuspended in PBS, and fluorescence was assessed by flow cytometry.

### Western blotting

Oral cancer cells (1 × 10^6^) were seeded in 100-mm dishes. After treatment with different concentrations of silibinin for 48 h, the cells were collected, washed with PBS, and lysed using Pro-Prep lysis buffer (Intron Biotechnology, Republic of Korea) for 40 min on ice. Proteins (30 μg) were separated by sodium dodecyl sulfate-polyacrylamide gel electrophoresis and transferred onto polyvinylidene difluoride membranes (EMD Millipore, Billerica, MA, USA). The membranes were blocked with 5% bovine serum albumin at room temperature (RT) for 1 h and incubated with primary antibodies at 4°C overnight. Membranes were then incubated with the corresponding secondary antibodies for 1 h at room temperature. Immunoblots were visualized with an ECL detection kit (GE Healthcare, Seoul, Republic of Korea) using a Davinch-K imaging system (Davinch-K, Seoul, Republic of Korea) 27.

### Ca9-22 xenograft tumors in mice

All animal experiments were performed in accordance with the guidelines and approval of Kyungpook National University. Male athymic nude mice (4-5 weeks) were purchased from Charles River Technology. The flanks of the mice were subcutaneously injected with Ca9-22 cells (5×10^6^ cells) suspended in 200 μl PBS to generate Ca9-22 xenografts. The mice were randomly separated into two groups (n = 6 mice per group) after six days of implantation, where one group was treated with silibinin (100 mg/kg body weight) dissolved in 5% DMSO and 10% Tween-20 in PBS and the second group was treated with vehicle only, with both groups being injected intraperitoneally three times per week for 32 days. The tumor volume and body weight were measured daily. Tumor volume was calculated using the following ellipsoid formula: tumor volume (mm^3^) (length × width × height × 0.52).

### Immunohistochemical analysis

Formalin-fixed and paraffin-embedded tumor tissues were cut into 4-m-thick serial slices. Tumor tissue slices were roasted at 60°C overnight, rehydrated with xylene and graded alcohols, and antigens were extracted via heat treatment in citrate buffer. (pH 6.0). The specimens were incubated overnight at 4°C with primary antibodies (Ki‐67, 1:200; p-p38, 1:200; mTOR, 1:200; cyclin D1, 1:200), followed by incubation with a biotin-conjugated secondary antibody (1:200) for 1 h at 37°C. Images were viewed under a microscope and processed using ImageJ (v. 4).

### Statistical analysis

All data are presented as mean ± SD from at least three separate experiments. A Student's *t*-test was used to establish significance. Statistical *p*-value < 0.05 was considered statistically significant.

## Results

### Silibinin inhibits oral cancer cell growth

**Figure 1:**
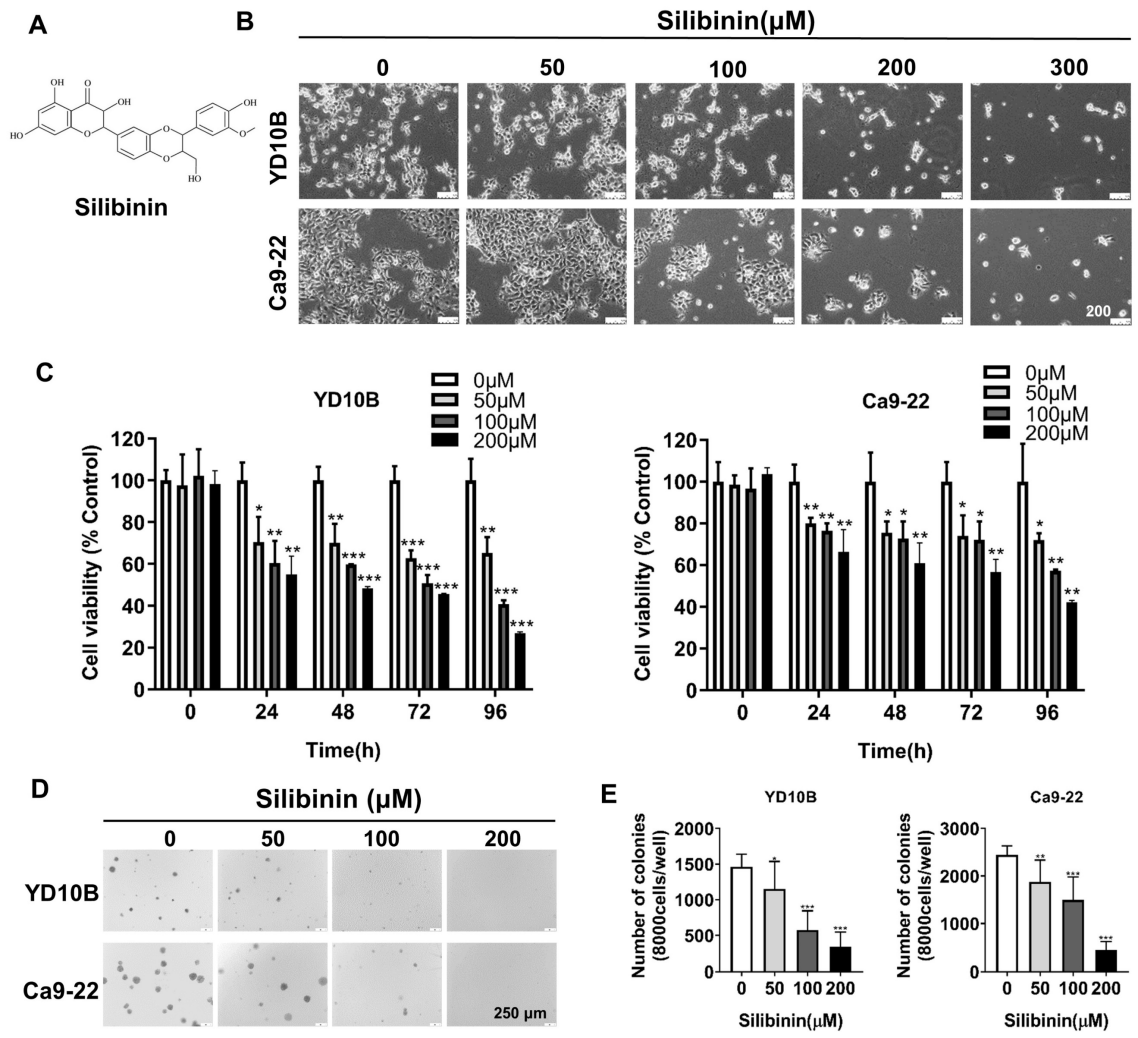
***Silibinin inhibits oral cancer cell growth.*** (A) Chemical structure of silibinin. (B) Morphology of YD10B and Ca9-22 cells after treatment with the indicated concentrations of silibinin for 48 h observed under a light microscope (magnification, 100×). (C) YD10B and Ca9-22 cells were treated with 0, 50, 100, and 200 μM silibinin for 0, 24, 48, 72, and 96 h. Cell viability was measured using CCK-8 assay. (D) Effects of silibinin on colony formation in YD10B and Ca9-22 cells (magnification, 50×). *p<0.05; ** p< 0.01; *** p< 0.001.

Figure 1A illustrates the chemical structure of the silibinin. YD10B and Ca9-22 cells were treated with 0, 50, 100, 200, and 300 μM silibinin for 48 h to explore the effects of silibinin on the proliferation of human oral cancer cells. As depicted in Figure 1B, the cell morphology changed to round and contracted, and cell density decreased following silibinin treatment, with most of the cells dying at a concentration of 300 μM. Thus, silibinin concentrations of 0, 50, 100, and 200 μM were chosen for subsequent experiments. The CCK-8 test was then used to determine cell viability after the treatment of oral cancer cells with 0, 50, 100, and 200 μM silibinin at different time points. Silibinin suppressed the growth of oral cancer cells in a dose- and time-dependent manner (Figure 1C). Furthermore, anchorage-independent cell growth tests revealed that silibinin significantly decreased the number and size of clones in both YD10B and Ca9-22 cells in a dose-dependent manner (Figure 1D, E). These results indicate that silibinin significantly suppressed the growth and colony formation of oral cancer cells.

### Silibinin inhibits oral cancer cell migration and invasion

**Figure 2:**
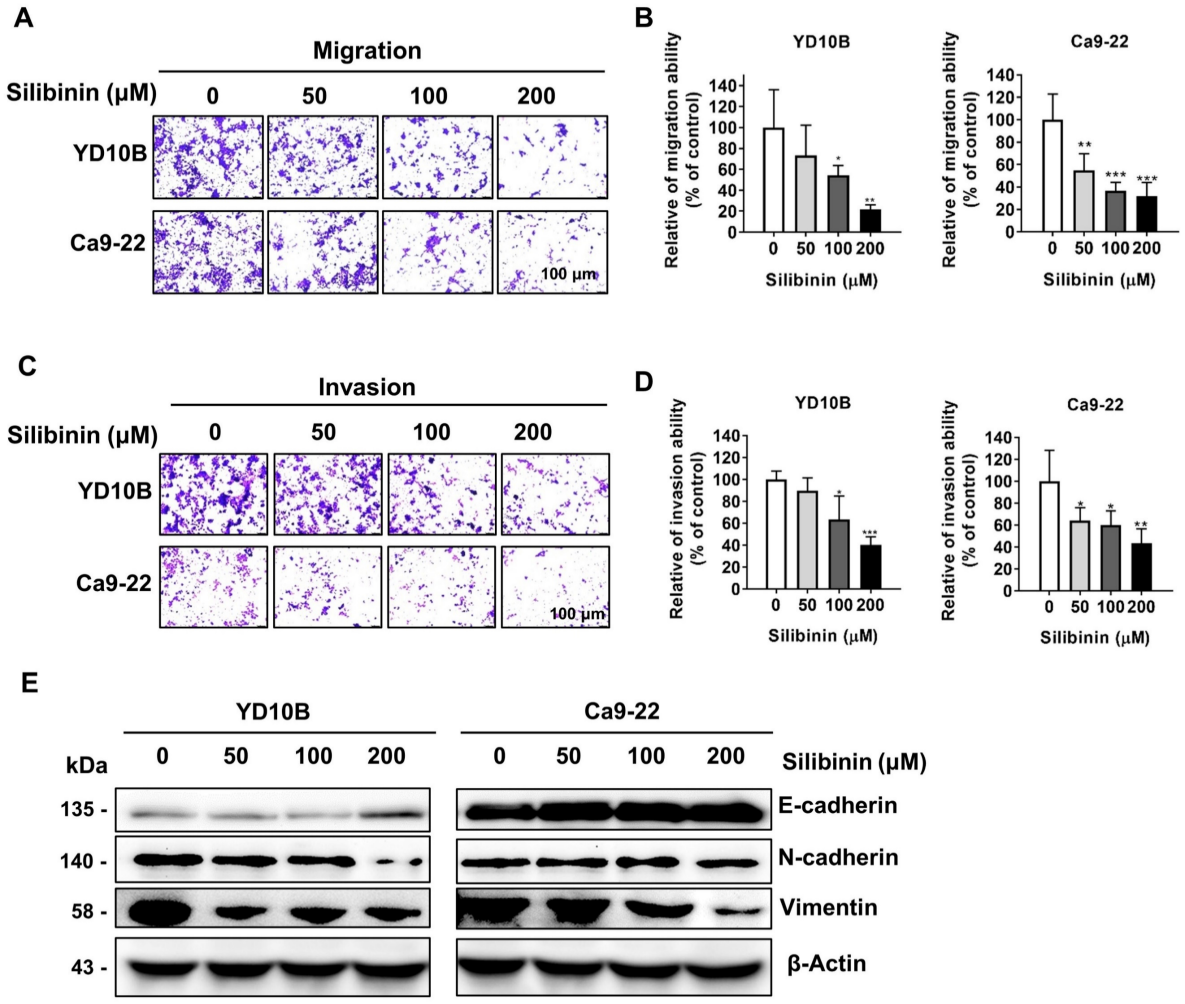
***Silibinin inhibits oral cancer cell migration and invasion.*** (A and B) Migration abilities of YD10B and Ca9-22 cells determined after treatment with 0, 50, 100, and 200 μM silibinin for 48 h. (C and D) Invasion abilities of YD10B and Ca9-22 cells determined after treatment with 0, 50, 100, and 200 μM silibinin for 48 h. (E) Western blot analysis of the expression of E-cadherin, N-cadherin, and vimentin in YD10B and Ca9-22 cells after treatment with 0, 50, 100, and 200 μM silibinin for 48 h. *p<0.05; ** p< 0.01; *** p< 0.001.

Metastasis is the main cause of cancer-related mortalities. A Transwell assay was performed to determine whether silibinin could decrease cancer cell metastasis. Silibinin effectively suppressed the migratory and invasive abilities of oral cancer cells in a dose-dependent manner (Figure 2A-D). Solid tumors could become more aggressive and more susceptible to spreading; and this is attributable to epithelial-mesenchymal transition (EMT), which also renders them more invasive 28, 29. The effect of silibinin on the expression of EMT marker proteins, such as E-cadherin, N-cadherin, and vimentin was further investigated. Immunoblotting results showed that silibinin downregulated N-cadherin and vimentin expression and upregulated E-cadherin expression in YD10B and Ca9-22 cells (Figure 2E). Together, these results indicate that silibinin inhibits the migration and invasion of oral cancer cells by suppressing the EMT.

### Silibinin induces cell cycle G_0_/G_1_ phase arrest in oral cancer cells

**Figure 3:**
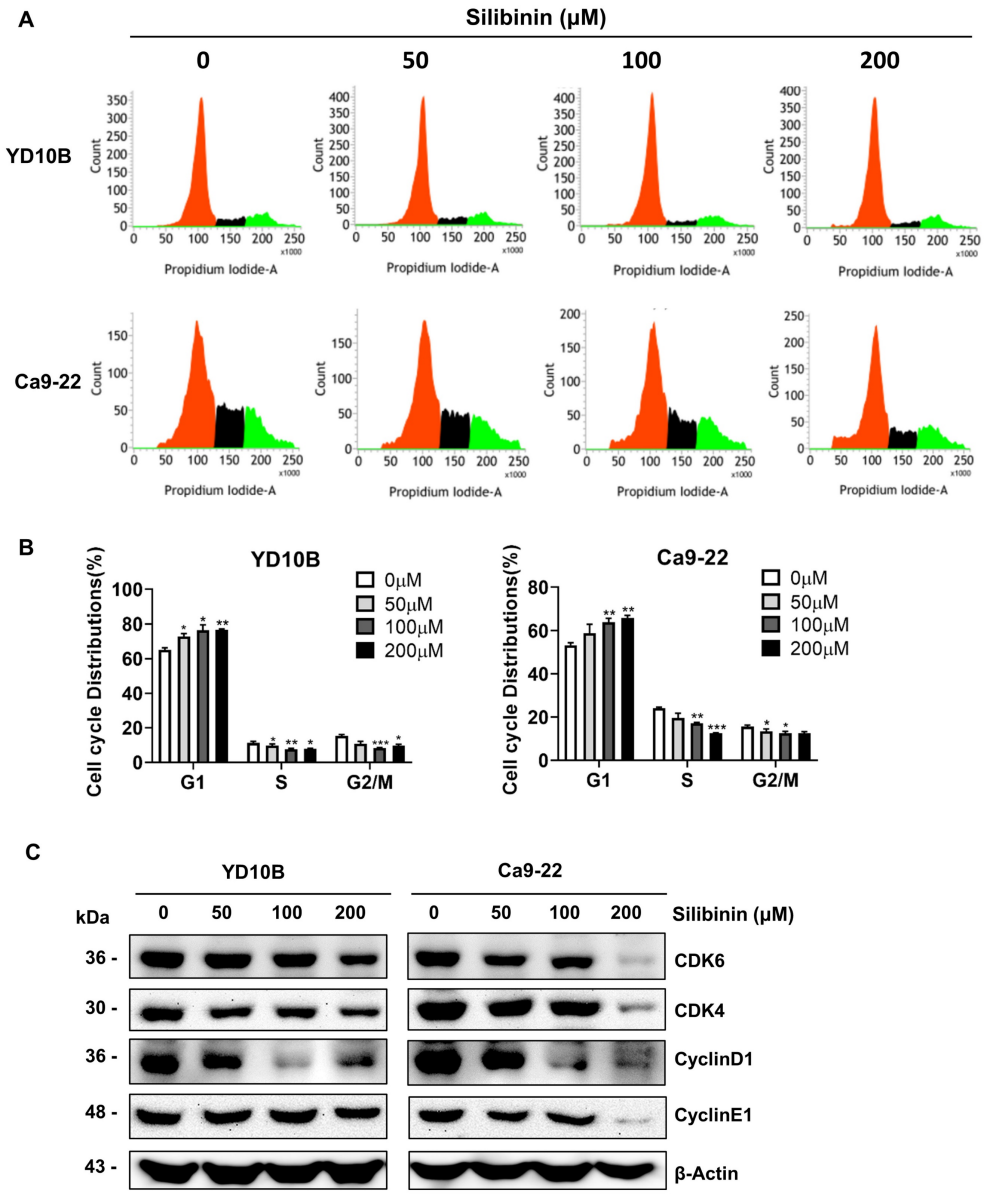
***Silibinin induces cell cycle G_0_/G_1_-phase arrest in oral cancer cells.*** (A) YD10B and Ca9-22 cells were treated with silibinin (0, 50, 100, and 200 μM) for 48 h and stained with PI. Cell cycle distribution was analyzed using flow cytometry. (B) Bar graph representing the proportions of cells in different phases. (C) The protein expression levels of cyclin-dependent kinase (CDK) 4, CDK6, cyclin D1 and cyclin E1 determined using western blotting. *p<0.05; ** p< 0.01; *** p< 0.001.

One of the approaches used in cancer chemotherapy is triggering cell cycle blockage, thus inhibiting tumor growth. To explore the mechanism by which silibinin prevents proliferation of oral cancer cells, a cell cycle assay was performed using flow cytometry. YD10B and Ca9-22 cells were treated with silibinin for 48 h, and the cell cycle phases were observed using flow cytometry. Silibinin significantly increased the proportion of cells in the G_0_/G_1_ phase in YD10B and Ca9-22 cells (Figure 3A and B). We examined the effects of silibinin on the expression of G_0_/G_1_ phase-associated proteins, including cyclin D1, cyclin E1, CDK4, and CDK6, using western blot analysis. As shown in Figure 3C, silibinin treatment downregulated the expression of cyclin D1, cyclin E1, CDK4, and CDK6 in the YD10B and Ca9-22 cells. These results indicated that the inhibition of cell proliferation by silibinin may be associated with the induction of G_0_/G_1_ phase arrest.

### Silibinin induces oral cancer cell apoptosis

**Figure 4:**
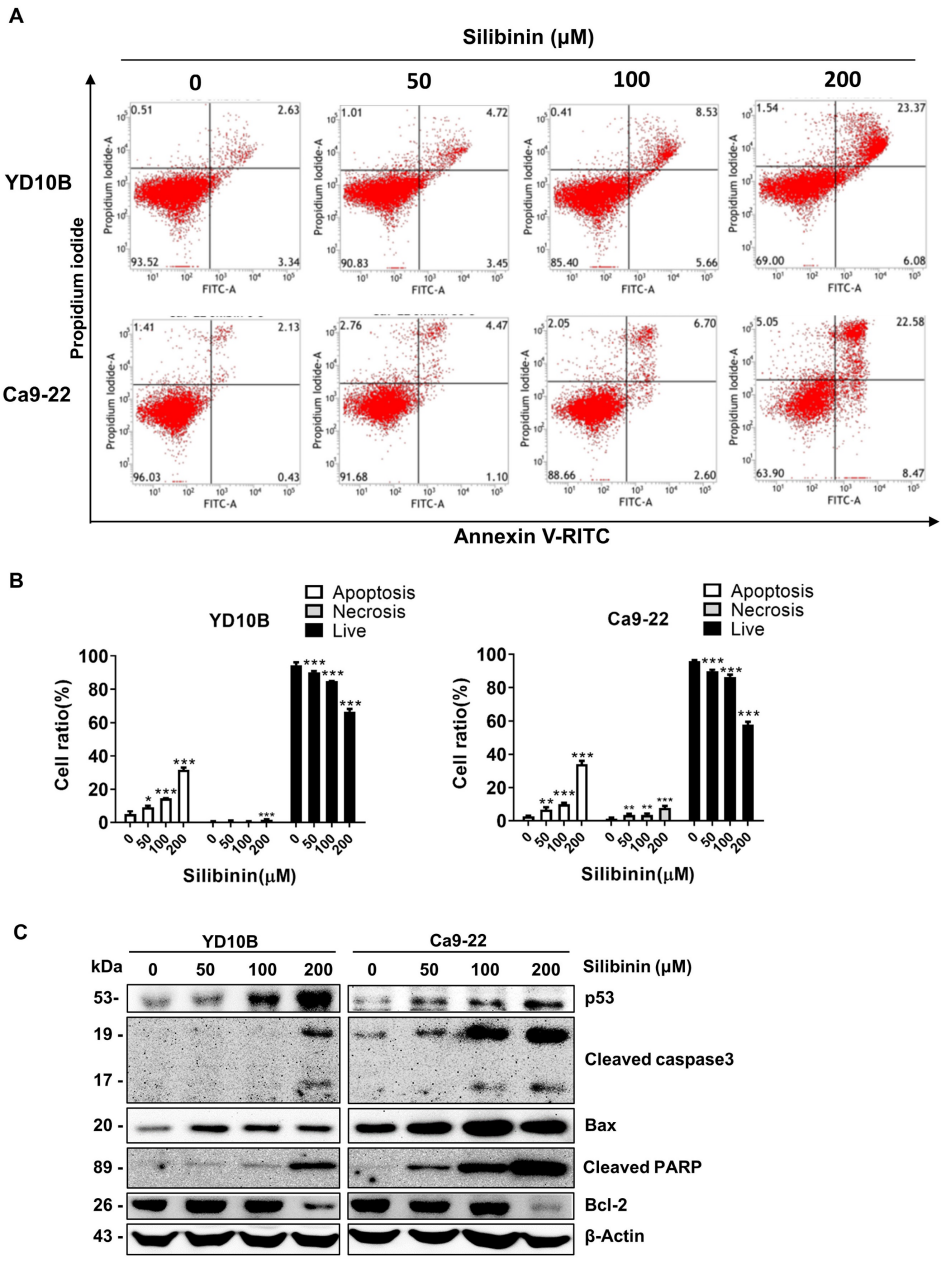
***Silibinin induces apoptosis in oral cancer cells.*** (A) YD10B and Ca9-22 cells were treated with silibinin (0, 50, 100, and 200 μM) for 48 h and stained with Annexin V-FITC/PI. Apoptosis was analyzed using flow cytometry. (B) Quantification of apoptotic cells after treatment with silibinin. (C) Protein levels of p53, cleaved caspase-3, cleaved PARP, Bax, and Bcl-2 determined using western blotting. *p<0.05; **p< 0.01; ***p< 0.001.

Targeting apoptosis has become a focus area in many cancer treatment strategies 30. To explore whether silibinin can induce apoptosis in oral cancer cells, we performed Annexin V-FITC/PI staining to quantify apoptotic cell populations after silibinin treatment. Based on these results, silibinin enhanced the fraction of apoptotic cells in a dose-dependent manner (Figure 4A and B). The accumulation of apoptosis-related proteins following silibinin therapy in oral cancer cells was investigated by western blotting. As shown in Figure 4C, silibinin significantly induced the expression of p53, cleaved caspase-3, cleaved PARP, and Bax, and downregulated the expression of the anti-apoptotic marker protein Bcl-2 (Figure 4C). These results indicate that silibinin induced mitochondria-mediated apoptosis in oral cancer cells.

### Silibinin induces ROS generation and activation of the JNK/c-Jun signaling pathway in oral cancer cells

**Figure 5:**
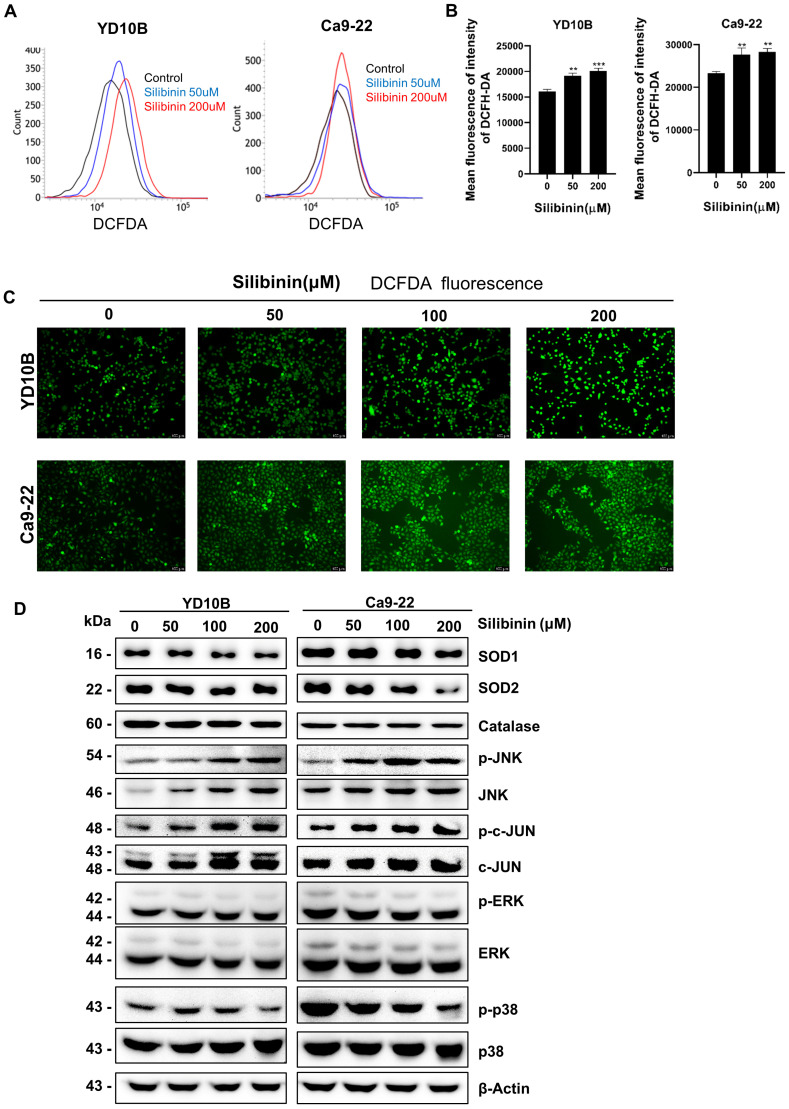
*** Silibinin induces reactive oxygen species (ROS) generation and activation of the JNK/c-Jun signaling pathway in oral cancer cells.*** (A) Cells were incubated with DCFH-DA for 30 min following treatment with silibinin for 24 h. ROS levels were measured using flow cytometry. (B) Histogram showing quantitative generation of ROS. (C) Cells were incubated with DCFH-DA for 30 min following treatment with silibinin for 24 h. ROS levels were visualized using fluorescence microscopy. Scale bar, 100 µm. (D) Protein levels of SOD1, SOD2, p-JNK, JNK, p-c-Jun, and c-Jun determined using western blotting.

ROS are also involved in apoptosis and cancer progression. Silibinin has been found to increases ROS generation and induces oxidative stress in several cancer cell lines 31. Therefore, we explored whether silibinin induces ROS production in oral cancer cells. YD10B and Ca9-22 cells were treated with 0, 50, and 200 μM silibinin for 24 h. ROS production was measured using a DCFH-DA probe and analyzed by flow cytometry. The results showed that ROS levels increased in response to silibinin treatment in YD10B and Ca9-22 cells (Figure 5A and B). Similar results were obtained for the immunofluorescence assay (Figure 5C). Antioxidant enzymes, such as mitochondrial superoxide dismutase (SOD) and catalase, play important roles in modulating the intracellular ROS balance 32, 33. Consequently, we studied the expression of SOD in oral cancer cells after treatment with silibinin and found that SOD1 and SOD2 expressions were consistently downregulated in oral cancer cells after treatment with silibinin (Figure 5D). Additionally, silibinin upregulated the expression of p-JNK and downregulated the expression of p-p38. The JNK signaling system regulates cell death caused by reactive oxygen and nitrogen species. 7. Western blotting was used to evaluate the expression of proteins involved in the JNK signaling pathway. The expression of phosphorylated (p)-JNK and (p)-c-Jun increased significantly in oral cancer cells after treatment with silibinin, indicating that the JNK/c-Jun pathway is activated by silibinin in oral cancer cells. Taken together, these results suggest that silibinin inhibits SOD expression, induces ROS production, and activates the JNK/c-Jun pathway in oral cancer cells.

### ROS scavenging decreases apoptosis in silibinin-treated oral cancer cells

**Figure 6:**
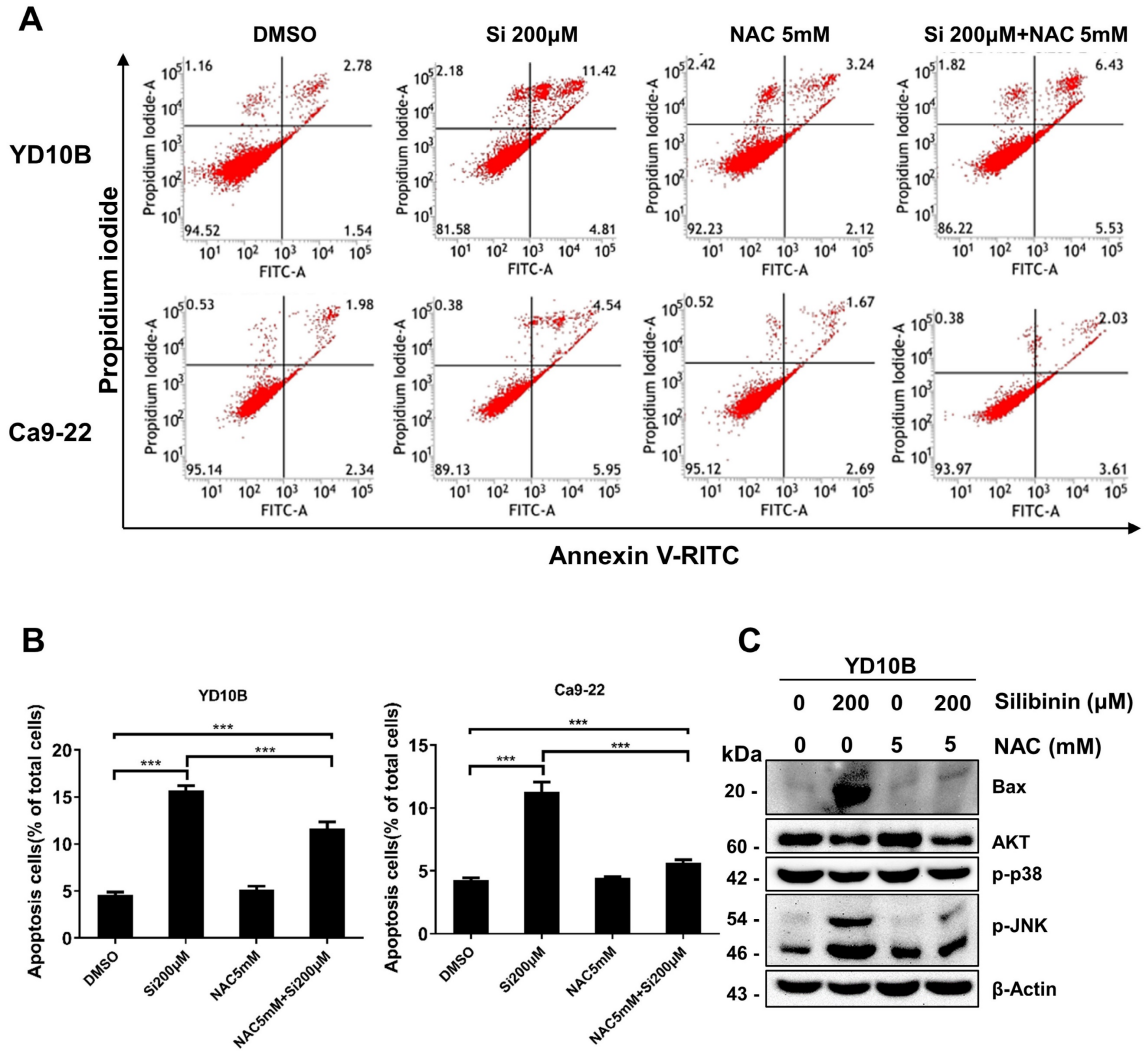
*** Scavenging of reactive oxygen species (ROS) decreases silibinin-induced apoptosis in oral cancer cells.*** (A) YD10B and Ca9-22 cells were pretreated with N-acetyl-l-cysteine (NAC) for 2 h, treated with or without silibinin (200 μM) for 24 h, and stained with Annexin V-FITC/PI. Apoptosis was analyzed using flow cytometry. (B) Quantification of apoptotic cells induced by silibinin and NAC. (C) Protein levels of Bax, AKT, p-p38, and p-JNK assessed using western blotting.

To confirm that silibinin induces apoptosis in oral cancer cells by regulating ROS levels, cells were pretreated with 5 mM NAC, a widely used ROS scavenger, for 1 h and treated with or without silibinin (200 μM) for 24 h. Interestingly, NAC significantly decreased silibinin-induced apoptosis (Figure 6A and B). Silibinin-induced upregulation of apoptosis-related proteins was diminished by NAC treatment (Figure 6C). Furthermore, NAC decreased the phosphorylation of JNK and upregulated the expression of p-ERK and p-p38 (Figure 6C). These results indicated that silibinin induces oral cancer cell death by inducing apoptosis and ROS generation.

### Silibinin suppresses Ca9-22 oral cancer tumor growth in a xenograft mouse model

**Figure 7:**
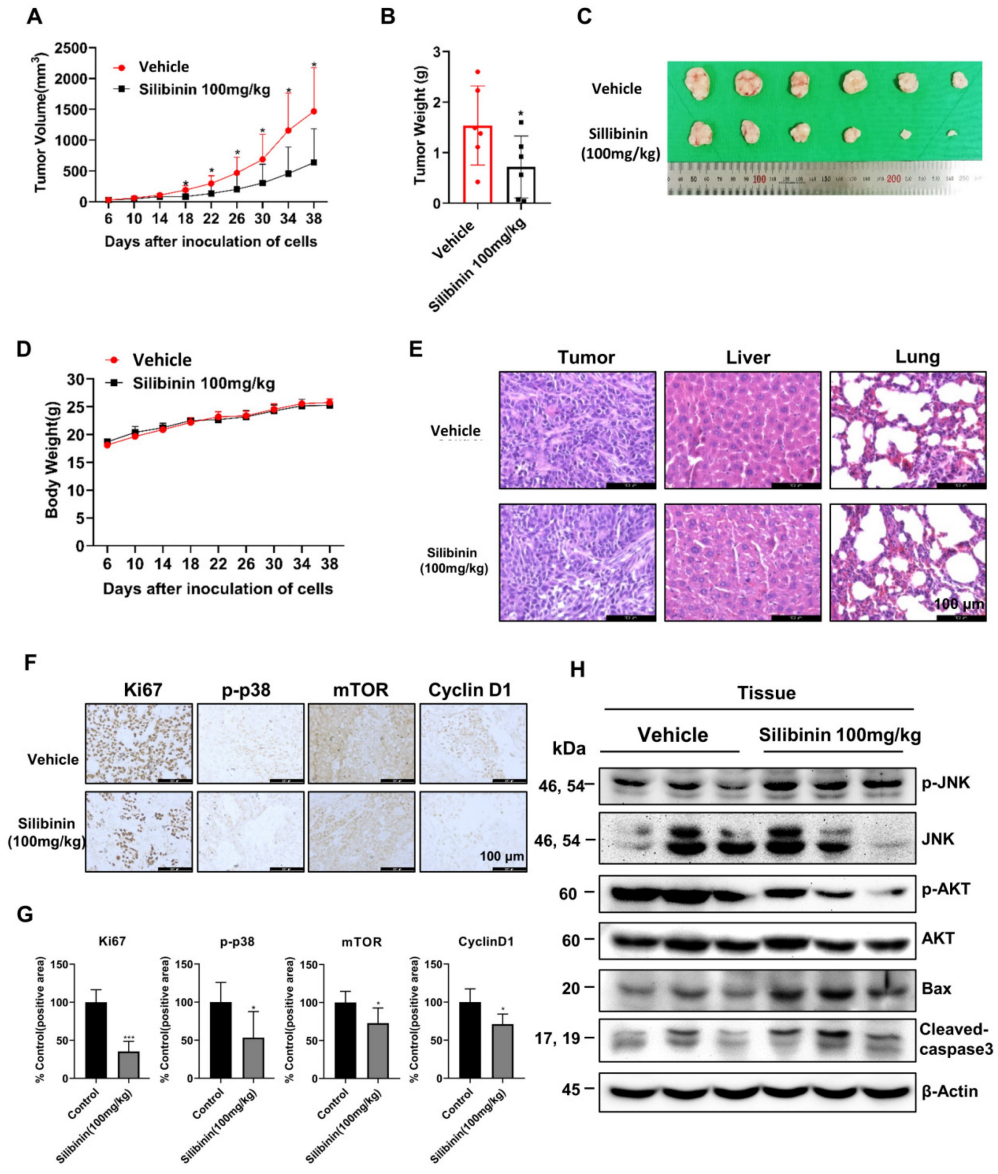
*** Silibinin suppresses oral cancer cell growth in a xenograft mouse model.*** Ca9-22 cells were injected subcutaneously into the right flank of BALB/c-nu mice, followed by intraperitoneal administration of the vehicle or silibinin (100 mg/kg) three times a week. (A) Tumor volume was measured every four days. (B) Tumor weight and (C) tumor mass were determined. (D) Body weights of mice were measured every four days. (E) Histology of the liver and lungs evaluated by H&E staining. (F) Ki67, p-p38, mTOR, and cyclin D1 levels were measured by immunohistochemistry. Representative images are presented. Scale bar, 100 µm. (H) Protein levels of p-JNK, JNK, p-AKT, AKT, Bax, and cleaved caspase 3 assessed using western blotting.

To investigate the anticancer properties of silibinin in vivo, we established a Ca9-22 xenograft model by injecting Ca9-22 cells subcutaneously into the flanks of nude mice. The mice were separated into two groups after 6 days and intraperitoneally injected with 100 mg/kg silibinin or vehicle thrice a week for 33 days. Silibinin significantly suppressed tumor growth and decreased tumor volume compared to the vehicle (Figure 7A-C). In addition, the body weight and histological structure of the liver of the silibinin-treated group showed no significant changes compared to those of the vehicle group, indicating that 100 mg/kg silibinin had no obvious toxicity in nude mice. To investigate the mechanism of action of silibinin in vivo, we performed immunohistochemistry (IHC) and immunoblotting assays. IHC results showed that the expression of the proliferation marker proteins Ki67, p-p38, mTOR, and cyclin D1 was inhibited in tumor tissues (Figure 7F). Western blotting results showed that the expression of p-JNK, Bax, and cleaved caspase-3 was upregulated, and p-AKT expression was downregulated. These results indicate that silibinin exhibits potent antitumor activity without significant toxicity and is therefore a potential drug for oral cancer treatment.

## Discussion

Although the prognosis of oral cancer has significantly improved owing to advances in surgery and multiagent chemotherapy, the overall 5-year survival rate remains unsatisfactory at approximately 65%. The low response rate of immunotherapy and resistance to targeted drugs result in oral cancer treatment failure. Innovative drugs and treatments are required to improve the outcome of patients with oral cancer. A growing number of studies has revealed that natural products possess the advantages of low toxicity and low drug resistance and can play a role in cancer prevention and treatment. Silibinin, the major flavonolignan isolated from *S. marianum,* has been reported as a promising therapeutic agent for different cancer types because of its potent antitumor effects and minimal toxicity 16, 18, 34, 35. However, the mechanism of action of silibinin in oral cancer remains unclear. In this study, we demonstrated that silibinin significantly inhibits oral cancer cell proliferation, migration, and invasion and induces apoptosis and cell cycle G_0_/G_1_ phase arrest. Furthermore, silibinin increased ROS generation and activated the JNK/c-Jun signaling pathway in these cells.

Silibinin (INN), also known as silybin is the major active constituent of silymarin, a standardized extract of the milk thistle seeds. Silymarin is well known to safe in humans at therapeutic doses and is well tolerated even at a high dose of 700 mg three times a day for 24 weeks 36. Also, there was no adverse reaction in after oral administration of silymarin at the dose of 140 mg three times daily for 28 days. Due to its low toxicity, it is also widely used as a treatment for liver-related diseases such as hepatitis C, nonalcoholic fatty liver disease, and other liver disease. Silibinin has anticancer and antiinflammatory effect on various disease such as lung injury and hepatocytes and is known to inhibit the cancer cell proliferation, and induced apoptosis by upregulation of ROS generation. Silibinin is well known to have several protective effects in liver 37, 38.Also, silibinin has anticancer effects by induction apoptosis and lipid peroxidation via ROS generation in liver cancer 39. It leads to MAPK signaling pathway related cell death in human choriocarcinoma cells and Hep-2 cells 40, 41. However, anticancer activity of silibinin in oral cancers related with ROS generation still remains unclear and requires further confirmation. Therefore, we identified whether silibinin have anti-cancer effects on oral cancer cells via ROS/MAPK signaling pathway.

Induction of apoptosis is a desirable alternative strategy for cancer treatment. p53 is known for its role in suppressing tumorigenesis, and p53-dependent apoptosis contributes to chemotherapy-induced cell death 42, 43. In this study, we found that p53 expression significantly increased in oral cancer cells after treatment with silibinin. Furthermore, Bcl-2 family proteins are critical regulators of apoptosis, and a high Bax to Bcl-2 ratio can cause the mitochondrial membrane potential to collapse, resulting in the release of cytochrome c and subsequent death. 44, 45. Our results indicated that silibinin downregulates Bcl-2 expression and upregulates Bax and cleaved caspase-3 expression. Furthermore, induction of cell cycle arrest is considered an effective strategy for cancer treatment. In this study, we investigated the cell cycle phase distribution using flow cytometry. The results revealed that the fraction of G0/G1 cells increased in a dose-dependent manner, indicating that silibinin induced G_0_/G_1_ arrest. Cell cycle progression is highly regulated by several cell cycle checkpoint proteins such as cyclins and CDKs. Among these, cyclins D and E together with CDK2, CDK4, and CDK6 play major roles in DNA replication and mitosis by regulating the G_0_/G_1_ phase 46, 47. We found that the expression levels of cyclin E1, cyclin D1, CDK4, and CDK6 were downregulated in the silibinin-treated oral cancer cells. These results indicated that silibinin suppressed oral cancer cell growth by inducing apoptosis and G_0_/G_1_ phase arrest.

Cancer cells are characterized by elevated aerobic glycolysis and high levels of oxidative stress. This increase in oxidative stress is usually caused by ROS accumulation. Elevated ROS levels can inhibit tumor growth through continuous cell cycle inhibition. Silibinin treatment induced ROS generation in oral cancer cells. In addition, upon the initiation of apoptosis, ROS levels increase due to the disruption of intracellular redox homeostasis and irreversible oxidation of lipids, proteins, or DNA, which in turn activates oxidative stress-induced apoptotic signaling 48. Therefore, we used NAC to scavenge ROS and found that the levels of apoptosis-related proteins were decreased in oral cancer cells. ROS play an important role in cell survival and death. Under normal physiological conditions, specific ROS levels assist in cell survival; however, excessive ROS levels cause cellular damage and apoptosis 49. Elevated levels of ROS in cancer cells compared to normal cells can be leveraged for cancer treatment. In contrast, several regularly used chemotherapy regimens produce large amounts of ROS to destroy cancer cells 4, 50. In this study, silibinin significantly increased ROS generation and JNK phosphorylation. It is widely assumed that mitochondria are the primary producers of cellular ROS. 51. ROS levels in cells affect many signaling pathways, including the JNK signaling pathway. We observed that silibinin therapy activated the JNK/c-Jun pathway. Inhibition of the invasion and metastasis of cancer cells is key to the success of cancer treatment. Numerous studies have shown that EMT is intimately linked to cancer cell invasion and metastasis. The expression of N-cadherin and vimentin increased EMT, whereas E-cadherin expression decreased it. In this study, we found that silibinin significantly inhibited the invasiveness and metastatic ability of oral cancer cells, upregulated E-cadherin expression, and downregulated N-cadherin and vimentin expressions. This suggests that silibinin exerts anticancer effects by inhibiting the invasion and metastasis of cancer cells. Distant metastasis of oral cancer is the main cause of death and recurrence. This study supports that silibinin have a positive impact on the survival rate of oral cancer patients thought suppresses the metastasis and proliferation and induced apoptosis by generating ROS in oral cancer via JNK signaling pathway. In conclusion, we revealed that silibinin significantly inhibited proliferation, migration, and invasion; induced apoptosis and G_0_/G_1_ phase arrest; induced ROS formation; and activated the JNK/c-Jun signaling pathway in oral cancer cells. Silibinin also significantly suppressed tumor growth, as was evident in the oral cancer cell xenograft mouse model. Therefore, our results indicate that silibinin is a potential anticancer agent that may be useful for oral cancer treatment.
